# Anti-tumor activity of phenoxybenzamine and its inhibition of histone deacetylases

**DOI:** 10.1371/journal.pone.0198514

**Published:** 2018-06-13

**Authors:** Mario A. Inchiosa

**Affiliations:** Departments of Pharmacology and Anesthesiology, New York Medical College, Valhalla, New York, United States of America; Peking University Health Science Centre, CHINA

## Abstract

The principal finding from this study was the recognition that the α-adrenergic antagonist, phenoxybenzamine, possesses histone deacetylase inhibitory activity. Phenoxybenzamine is approved by the United States Food and Drug Administration for the treatment of hypertensive crises associated with tumors of the adrenal medulla, pheochromocytomas. It has several “off label” indications relative to its capacity to relax vascular smooth muscle and smooth muscle of the urogenital tract. The drug also has a long history of apparent efficacy in ameliorating, and perhaps reversing, the severe symptoms of neuropathic pain syndromes. Our interest in this feature of the drug relates to the fact that certain types of neuropathic pain, in particular complex regional pain syndrome, demonstrate a proliferative nature, with the capacity to spread from an injured limb, for example, to a non-injured limb and perhaps to essentially the entire body. Sensory neuronal sprouting in the spinal cord has been observed under conditions where there is a high sensory input from painful stimuli. Searches of gene expression signatures in the BroadBuild02 Molecular Signature Database using their connectivity map software suggested that phenoxybenzamine may have histone deacetylase inhibitory activity. Studies by others have reported inhibitory effects of phenoxybenzamine on growth, invasion and migration of human tumor cell cultures and, in one study, inhibition of tumor expansion in animal experiments. Inhibitory effects on human tumor cell cultures are also reported in the present study. Phenoxybenzamine was also found to have histone deacetylase inhibitory activity; histone deacetylase isoforms 5, 6, and 9 were the most sensitive to inhibition by phenoxybenzamine. The importance of elevated levels of these isoforms as biomarkers of poor prognosis in human malignant disease, and the recognized suppression of tumor growth that may accrue from their inhibition, opens consideration of possible translation of phenoxybenzamine to new clinical applications. This might be facilitated by the fact that phenoxybenzamine is already an approved drug entity. There appears to be no previous report of the activity of phenoxybenzamine as a histone deacetylase inhibitor.

## Introduction

**Fig 1:**
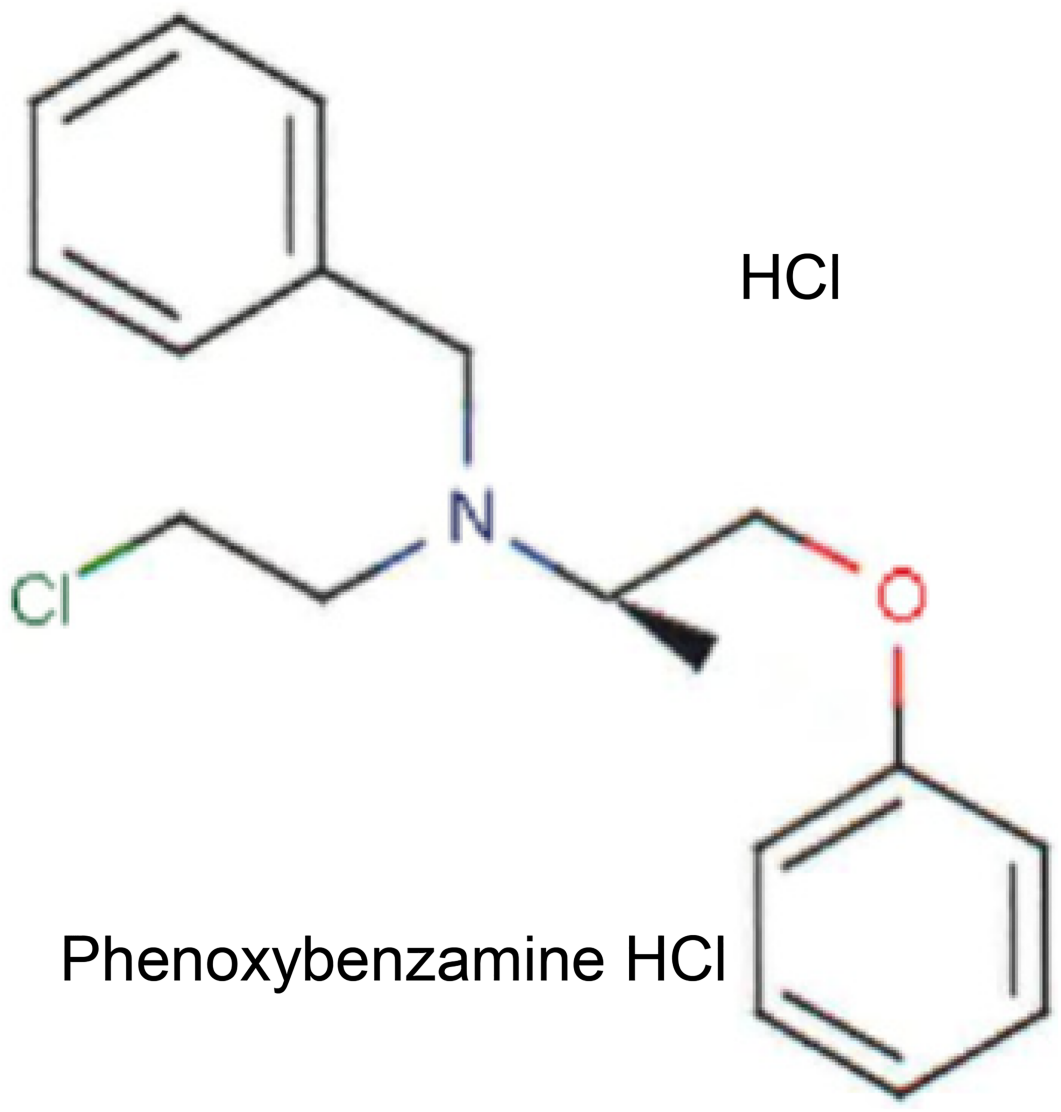
Chemical structure for phenoxybenzamine HCl.

Phenoxybenzamine (PBZ) is classified chemically as a haloalkylamine (Fig 1). It was approved in 1953 by the United States Food and Drug Administration (FDA) for the treatment of hypertensive emergencies, in particular for the control of blood pressure in patients secreting large quantities of epinephrine and norepinephrine from tumors of the adrenal medulla, termed pheochromocytomas. Its United States proprietary name is Dibenzyline, but generic preparations are available. The drug forms covalent bonds with α_1_- and α_2_—adrenergic receptors resulting in a long-lasting non-competitive antagonism of these receptors. The drug has additional (non-FDA-labeled) indications related to its relaxing effects on vascular smooth muscle in peripheral vascular diseases and the smooth muscle of the urogenital tract (http://www.ahfsdruginformation.com) [1].

Interest in the potential anti-proliferative activity of PBZ evolved from observations of its apparent efficacy in the treatment of the chronic neuropathic pain syndrome, Complex Regional Pain Syndrome (CRPS), which was previously termed Reflex Sympathetic Dystrophy. An initial study was based on the known non-competitive (irreversible) alpha-adrenergic antagonist activity of PBZ against the mediators of the sympathetic nervous system, norepinephrine and epinephrine, in an attempt to antagonize the presumed sympathetic dystrophy [2,3]. Apparent efficacy was observed in this small human study. This first study employed an investigational intravenous formulation of PBZ, but further work was precluded by the lack of availability of a stable intravenous formulation; there is still no preparation available today.

In a subsequent series of case reports with the FDA-approved oral preparation of the drug, there was again observed apparent efficacy for treatment of CRPS and an hypothesis was presented for possible mechanisms of action in this syndrome [4]. It was noted in this report that in addition to its alpha-adrenergic antagonist activity, PBZ was also a potent non-competitive (irreversible) inhibitor of calmodulin, and integrated this activity into its efficacy in CRPS. An anti-inflammatory/immunomodulatory mechanism of action for the drug was included in a later review [5].

A study by Chang et al. [6] added considerable support to the possible therapeutic value of PBZ for the treatment of CRPS. They identified patterns of genes that were differentially expressed in the Complete Freund’s Adjuvant (CFA) animal model of CRPS. This model results in tactile allodynia and thermal hyperalgesia. Gene map arrays from RNA extracts of the L4 and L5 dorsal root ganglia of rats that had received intraplantar CFA for 4 days revealed more than 100 genes that were significantly (> 1.5 fold; p ≤ 0.05) up- or down-regulated. The genes involved immune function, inflammatory response, and neuronal growth. The total pattern of gene changes was considered to be a gene “signature” of the CFA model. Their search of the Broad Build02 database [7] for pharmacologically active compounds that had strong inverse gene signatures in relation to that of the CFA pathology yielded PBZ as one of five compounds with the strongest inverse matches of gene expression. The inverse signature suggested possible therapeutic value in this pain syndrome. They also demonstrated that PBZ was equipotent with the nonsteroidal anti-inflammatory drug, naproxen, in reversing the heightened pain sensitivity of the CFA-affected animals [6].

The relevance of these earlier investigations to this present study relates to the rather remarkable fact that CRPS appears to have a substantial proliferative component in regard to both pain severity and expansion of anatomical involvement [8–10]. Thus, it has been observed that there is an anatomical spread of the pain and associated symptoms in the vast majority of cases [11]. The spread may be related to reports of sensory neuronal dendritic sprouting to the dorsal horn of the spinal cord when there is high sensory input [12–14]. It is this proliferative activity in the syndrome that led to the investigation of the possible anti-proliferative activity of PBZ, particularly in relation to possible inhibitory effects on histone deacetylase (HDAC) activity.

The importance of the epigenetic modulation of gene expression by HDACs and histone acetyltransferases (HATs) does not need to be reviewed here. The balance between the actions of HDACs and HATs is extremely complex and the interaction of these effects relates to many examples of dysregulation of gene expression that are associated with malignant transformation and expansion, and autoimmunity [15]. The initial recognition of PBZ as a potential inhibitor of HDAC was revealed through searches of the BroadBuild02 (Broad Institute Harvard/Massachusetts Institute of Technology) Molecular Signature Database (MSigDB) with the use of their Connectivity Map (CMap) software [7,16]. CMap has gained a broad role in genomic investigations relative to repurposing of existing drugs, hypothesis testing, mechanisms of action and lead discoveries [17]. The co-sorting of PBZ in CMap with known HDAC inhibitors was followed up with tests of anti-proliferation of human tumor cell lines, and then with direct tests for inhibition of HDACs by PBZ.

The chemical structure of PBZ (Fig 1) does not appear to have any particular features that are similar to known HDAC inhibitors. In fact, the structure of PBZ is distinguished from known inhibitors by the fact that it is a haloalkylamine, with a substituted chlorine atom; 21 congeners of PBZ have been described, all with chorine as the halogen substitution [18]. These congeners showed varying potencies in regard to a common property of inhibition of uptake of catecholamines into presynaptic sympathetic nerve terminals in the isolated rat heart, and at extraneuronal uptake processes; none of these compounds have been investigated in the present study.

## Materials and methods

### PBZ searches on the Harvard-MIT molecular signature database

The open web-based platforms of BroadBuild02 CMap (www.broad.mit.edu/cmap) and MSigDB (www.broadinstitute.org/gsea/msigdb) were used for all of the genomic expression searches. Only simple registration is necessary to access these databases and the associated CMap software. This database consists of approximately 7,000 gene expression profiles for 1309 FDA-approved drugs and other compounds of interest as gene expression modulators. The profiles (‘instances,” in the BroadBuild terminology) are based almost entirely on the effects of compounds on MCF7 (breast cancer), PC3 (prostate cancer) and HL60 (leukemia) cells. The database includes 3 gene expression profiles for PBZ in MCF7 cells and one profile in PC3 cells. A common core gene expression set resulting from the effects of three HDAC inhibitors (suberanilohydroxamic acid (SAHA); trichostatin A (TSA) and MS-275) in human breast cancer cells (MDA 468 and MDA 435) and human bladder cancer cells (T24) [19] was first evaluated for the relative association of the 4 PBZ gene expression profiles with known HDAC inhibitors. CMap also provided a measure of the relative selectivity of PBZ for this common gene expression signature compared with other gene signatures where PBZ was found to have higher gene enrichment scores than in the common core set noted above [19]. Enrichment scores represent a relative measure of the correspondence between a gene signature of interest (e.g. a biological state) and a calculated composite measure of the 4 gene expression profiles of PBZ in CMap [20].

### Effects of PBZ on human tumor cell proliferation

The results of these studies were included in an application for the screening of PBZ in the National Cancer Institute NCI60 screening program (https://dtp.cancer.gov/discovery_development/nci-60); they measured its effects on growth in cultures of 60 human tumors. This initial screen was conducted at only one drug concentration, 10 μM. We supplied the United States Pharmacopeial reference standard of PBZ-hydrochloride in powder form. The NCI60 standard procedure for dissolution of a synthetic molecule like PBZ involved initial dilution in 9:1 dimethyl sulfoxide (DMSO): glycerol at a concentration of 4 mM; the solution was then diluted 1:200 with complete culture medium (RPMI 1640 medium containing 5% fetal calf serum (FCS), 2 mM L-glutamine and 50 μg/ml gentamicin to give a final concentration of 20 μM; 100 μl of the drug-medium mixture was added to 100 μl of the cell cultures already in complete medium. The microtiter plates were incubated for 48 hr at 37°C, 5% CO_2_, 95% air, and 100% relative humidity (see S1 Appendix for further details). An important methodological point gained from an initial study was the observation that PBZ had essentially no biological effect if initially dissolved in DMSO.

Since PBZ has adequate aqueous solubility in the NCI60 assay conditions, their laboratory changed the initial solution and dilution steps as follows: “we solubilized in water (to 0.004M), transferred 75 μL to a dilution plate and sealed under argon in 20 minutes or less, and that dilution plate was then diluted by adding 15 mL of media (in no more than 3 hrs from initial solubilization), mixed and then immediately transferred 1:1 (100 μL into 100 μL) into growing cells.” Anti-tumor activity was measured with the Sulforhodamine B assay (BioVision, Inc., Milpitas, CA). PBZ demonstrated biological activity under these assay conditions. The reason for the lack of activity after dissolution in DMSO has not been pursued further, but DMSO was eliminated in all subsequent assay systems.

Further studies with varying drug concentrations on selected human cell lines, in most cases those studied in the NCI60 screen, were conducted by Oncotest GmbH laboratories, Freiburg, Germany; Oncotest is a division of Charles River Laboratories. The dissolution procedure for PBZ and the cell culture assays conditions were similar to those at the NCI except that the culture medium contained 10% FCS and the incubation period was 72 hours; anti-tumor activity was determined with the CellTiter-Blue^®^ Cell Viability Assay, Promega, Mannheim, Germany. The 50% Inhibitory (IC_50_) concentrations were determined. The results are the average of duplicate assays. Paclitaxel was included as a reference compound to confirm the integrity of the assays. See S1 Appendix for further details.

#### Effects of PBZ on histone deacetylase activity

Assays of the effects of PBZ on the deacetylase activity of HDACs 1 thru 11 were carried out by BPS Bioscience Laboratories, San Diego CA. PBZ was dissolved in isotonic phosphate-buffered saline (PBS) pH 7.4, at a concentration of 100 μM in our laboratory. It was shipped in this solution to BPS Bioscience Laboratories. The 100 μM stock solution was diluted with PBS for IC_50_ assays at the time of assay. The drug was pre-incubated for 1 hour at 37°C with the HDAC enzymes before addition of substrate. No DMSO or bovine serum albumin was present in the assays. Fluorogenic acetylated lysine peptides, according to the laboratories standard conditions and concentrations, were used as substrates (as noted in the results); fluorescence is suppressed until the acetyl group is removed by enzymatic activity, allowing a fluorescent measure of deacetylase activity. The assays were completed during the day following their initial dissolution in PBS. IC_50_ values for PBZ and SAHA or TSA (positive controls) were determined for each HDAC. See S2 Appendix for further details.

## Results

### PBZ searches on the Harvard-MIT molecular signature database

The output from CMap identified a relatively potent gene enrichment score [20] for PBZ in relation to a common core gene set resulting from inhibition by several HDACs in the human breast cancer cells, MDA 468 and MDA 435, and in cells of T24 human bladder cancer [19]. The gene enrichment score for PBZ was 0.818, p = 0.00193; it ranked 14th in relation to an enrichment score of 0.974 for MS-275, 0.973 for SAHA, 0.942 for scriptaid, and 0.895 for TSA, all established HDAC inhibitors (Table 1). All of the remaining compounds listed in Table 1, with the exception of pimozide (an anti-psychotic drug), have established or investigational anti-proliferative mechanisms of action.

**Table 1:** Rank order of gene enrichment score for phenoxybenzamine in relation to HDAC inhibitors.

CMap Name	Enrichment Score	p value
**MS-275**	**0.974**	**0.00000**
**vorinostat**	**0.973**	**0.00109**
**rifabutin**	**0.971**	**0.00004**
**scriptaid**	**0.942**	**0.00024**
**5707885**	**0.913**	**0.00004**
**withaferin A**	**0.896**	**0.00010**
**trichostatin A**	**0.895**	**0.00000**
**suloctidil**	**0.888**	**0.00016**
**emetine**	**0.888**	**0.00016**
**ivermectin**	**0.858**	**0.00012**
**pimozide**	**0.852**	**0.00072**
**cephaeline**	**0.848**	**0.00018**
**cicloheximide**	**0.830**	**0.00127**
**phenoxybenzamine**	**0.818**	**0.00193**

From Broad Institute Connectivity Map, in relation to gene signatures from reference [19] for bladder and breast cancers.

When the “selectivity” option of CMap was used to probe the relation of PBZ to other gene signatures in the MSigDB, the output produced a considerable list of gene enrichment scores for PBZ that were greater than that presented in relation to the Glaser [19] signature, and gave further evidence of possible association with various involvements in histone modifications (Table 2). A total of 24 gene expression signatures were identified where PBZ showed comparable or greater gene enrichment scores than TSA or SAHA. There were four groups of signatures that were disproportionately represented: Ultraviolet radiation of the epidermis in the low wavelength range (UVC); cytomegalovirus replication processes (CMV); ultraviolet radiation in the intermediate wavelength range (UVB); and gene signatures resulting from exposure of colon cancer cells to HDAC inhibitors.

**Table 2:** CMap SPECIFICITY: Phenoxybenzamine scored better with the following MSigDB gene sets than in the Glaser gene set (Table 1), and is compared with scores for TSA and SAHA with the same gene sets.

PBZ Enrichment	
Score	Gene Set
**0.998**	**UVC_TTD_8HR *(TSA 0*.*472; SAHA 0*.*723)***
**0.995**	**UVC_XPCS_8HR *(TSA 0*.*669; SAHA 0*.*888)***
**0.994**	**UVC_TTD-XPCS_COMMON**
**0.994**	**AS3_HEK293 *(TSA 0*.*485; SAHA 0*.*674)***
**0.993**	**UVC_LOW_ALL**
**0.993**	**UVC_XPCS_ALL *(TSA 0*.*680; SAHA 0*.*969)***
**0.991**	**UVC_TTD_ALL *(TSA 0*.*489; SAHA 0*.*874)***
**0.990**	**CMV_UV- CMV_COMMON_ HCMV_6HRS *(TSA 0*.*614; SAHA 0*.*863)***
**0.989**	**ET743_HELA *(TSA 0*.*540; SAHA 0*.*642*)**
**0.986**	**CMV_HCMV_6HRS**
**0.986**	**UVC_XPCS_4HR *(TSA 0*.*470; SAHA 0*.*657)***
**0.982**	**CHEN_HOXA5_TARGETS**
**0.982**	**CMV_8HRS *(TSA 0*.*588; SAHA 0*.*615)***
**0.978**	**ZHAN_MM_MOLECULAR_ CLASSI**
**0.977**	**CMV-UV_HCMV_6HRS *(SAHA 0*.*937)***
**0.975**	**MIDDLEAGE**
**0.974**	**CMV_HCMV_TIMECOURSE_10HRS *(SAHA 0*.*663)***
**0.972**	**UVC_TTD_4HR *(SAHA 0*.*618)***
**0.970**	**CMV_HCMV_TIMECOURSE_ALL *(TSA 0*.*478; SAHA 0*.*785)***
**0.969**	**OXSTRESS_BREASTCA**
**0.963**	**SHEPARD_CRASH_AND_BURN_MUT_VS_WT**
**0.963**	**CROONQUIST_IL6_RAS**
**0.962**	**UVC_HIGH_ALL *(TSA 0*.*638; SAHA 0*.*794)***
**0.961**	**UV-CMV_UNIQUE_HCMV_6HRS**
**0.960**	**HBX_HCC**
**0.954**	**STRESS_ARSENIC_SPECIFIC**
**0.942**	**UVB_NHEK2 *(TSA 0*.*598; SAHA 0*.*795)***
**0.941**	**BRCA1_OVEREXP_PROSTATE**
**0.935**	**GAY_YY1 *(TSA 0*.*608; SAHA 0*.*786)***
**0.925**	**KNUDSEN_PMNS**
**0.922**	**ELONGINA_KO**
**0.919**	**ET743_SARCOMA_24HRS *(TSA 0*.*554; SAHA 0*.*755)***
**0.916**	**SMITH_HTERT**
**0.912**	**UVB_NHEK1*(TSA 0*.*559; SAHA 0*.*753)***
**0.909**	**HDACI_COLON_BUT24HRS *(TSA 0*.*885; SAHA 0*.*976)***
**0.903**	**MUNSHI_MM_VS_PCS**
**0.896**	**NEMETH_TNF**
**0.893**	**CANTHARIDIN**
**0.891**	**KANNAN_P53**
**0.881**	**TPA_SENS_EARLY**
**0.876**	**WERNERONLY_FIBRO**
**0.872**	**CMV_HCMV_TIMECOURSE_12HRS**
**0.869**	**CAMPTOTHECIN_PROBCELL *(TSA 0*.*514; SAHA 0*.*726)***
**0.869**	**IGFR_IR**
**0.868**	**HDACI_COLON_SUL**
**0.866**	**PARP_KO *(TSA 0*.*574; SAHA 0*.*769)***
**0.862**	**TAKEDA_NUP8_HOXA9_16D**
**0.855**	**HEDVAT_ELF**
**0.850**	**HDACI_COLON_BUT12HRS *(TSA 0*.*882; SAHA 0*.*980)***
**0.844**	**GH_AUTOCRINE**
**0.836**	**PENG_LEUCINE*(TSA 0*.*523)***
**0.823**	**HDACI_COLON_TSA *(TSA 0*.*716; SAHA 0*.*889)***

In all of the gene signatures where comparisons were possible among the enrichment scores for PBZ, TSA and SAHA, the scores for SAHA were greater than for TSA. SAHA enrichment was greater than PBZ for only 3 gene signatures, HDACI_COLON _BUT24 HRS, HDACI_COLON_BUT12HRS, and HDACI_COLON_TSA; TSA was greater than PBZ for only the HDACI_BUT12HRS signature.

### Effects of PBZ on human tumor cell proliferation

**Fig 2:**
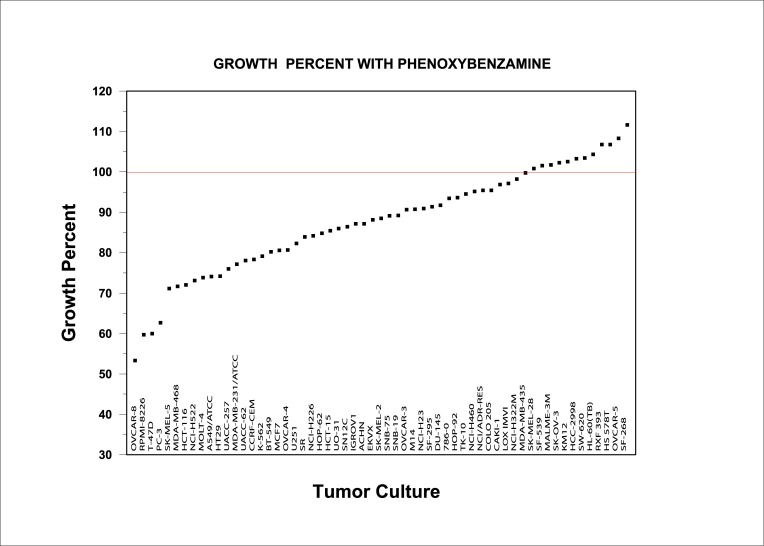
Effects of PBZ on cellular proliferation in the NCI60 human tumor culture screen.

The CMap results formed the basis for an application to the NCI60 screening program for anti-tumor efficacy. The results from that screen for PBZ at a 10 μM concentration are presented in Fig 2. PBZ showed a range of inhibitory activity on many of the cultures in NCI60 screen.

Several of the cultures that showed greater anti-tumor sensitivity to PBZ were studied further at the laboratories of Oncotest GmbH (Table 3). There was uniformly more inhibitory activity to PBZ when tested with 5% FCS for 48 hours (NCI60 assays) than with 10% FCS for 72 hours (Table 3).

**Table 3:** Comparison of percent growth in several cell cultures in the presence of 10 μM phenoxybenzamine with 5% and 10% fetal calf serum and different incubation periods.

Cell line		Tumor Class	Percent cell growth in NCI60 screen with 5% FCS (48 hr incubation)	Percent cell growth in Oncotest screen with 10% FCS (72 hr incubation)
LXFA	NCI-H522	Non-Small-cell Lung	73	108
MAXF	T47D	Breast	60	97
MEFX	UACC-257	Melanoma	76	107
MMXF	RPMI 8226	Leukemia	60	98
OVXF	OVCAR-8	Ovarian	53	90
PRXF	PC-3	Prostate	63	86

**Fig 3:**
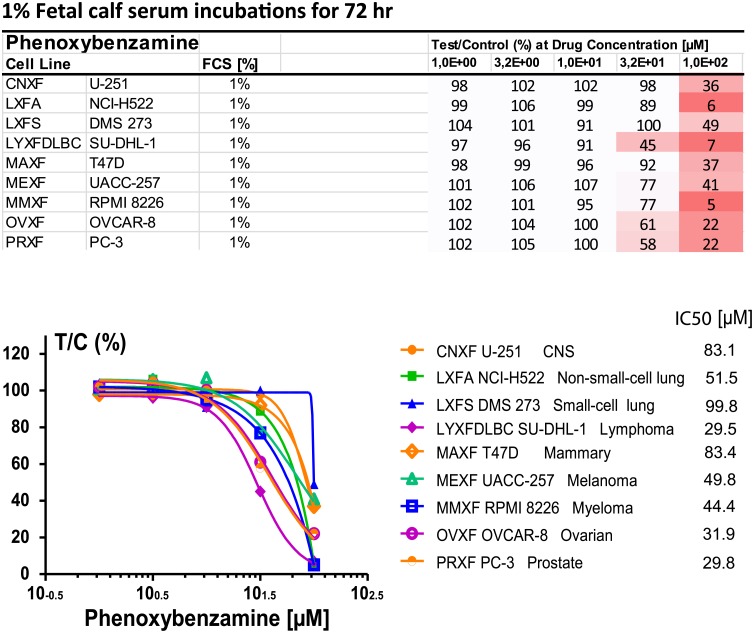
Effects of PBZ on cellular proliferation in 9 human tumor cell cultures of Oncotest laboratories with a medium containing 1% FCS. Gradations of red color intensity in the list of cell lines increase with increasing degree of inhibition.

An additional study was conducted with 1% FCS (Fig 3). This study included 3 additional cell cultures that were not part of the NCI60 screen: CNXF U-251, a CNS tumor; LXFS DMS 273, a small-cell lung tumor; and LYXF DLBC SU-DHL-1, a lymphoma. The differences in these assay conditions and their possible interactions were not systematically studied further. Fig 3 includes the IC_50_ values for PBZ for each of the 9 cell cultures that were tested at 1% FCS.

A summary of the 3 trials by Oncotest laboratories (Table 4) gives further evidence of the effect of FCS concentration on PBZ inhibition, in particular in the assays with PBZ at 100 μM concentration. Some evidence is also seen at a PBZ concentration of 32 μM. It may be speculated that non-specific binding of PBZ to protein may have reduced the free concentration of PBZ available for cellular interaction.

**Table 4:** Summary of PBZ effects on human tumor cell culture proliferation at two concentrations of FCS.

Phenoxybenzamine				Test/Control (%) at Drug Concentration [uM]										
Cell Line	Study No.	ECS	Exp No	1,0E-03	3,2E-03	1,0E-02	3,2E-02	1,0E-01	3,2E-01	1,0E+00	3,2E+00	1,0E+01	3,2E+01	1,0E+02
CNXF U-251	P495A	10%	QA1489-P1456245-5	-	85	90	93	90	93	92	94	103	118	98
	-	-	-	-	-	-	-	-	-	-	-	-	-	-
	P495C	1%	RA0214-P1537009-4	-	-	-	-	-	-	98	102	102	98	36
LXFA NCI-H522	P495A	10%	QA1490-P1456251-5	-	76	81	82	96	98	99	107	108	110	14
	P495B	10%	QA1971	100	-	100	-	100	-	100	100	93	97	29
	P495C	1%	RA0214-P1537009-5	-	-	-	-	-	-	99	106	99	89	6
LXFS DMS273	P495A	10%	QA1489-P1456245-6	-	83	75	74	81	80	76	87	82	100	105
	-	-	-	-	-	-	-	-	-	-	-	-	-	-
	P495C	1%	RA0215-P1536458-4	-	-	-	-	-	-	104	101	91	100	49
LYXF SU-DHL-1	P495A	-		-	-	-	-	-	-	-	-	-	-	-
	P495B	10%	QA1976	100	-	100	-	100	-	98	100	100	94	0
	P495C	1%	RA0218-P1537021-4	-	-	-	-	-	-	97	96	91	45	7
MAX T47D	P495A	10%	QA1491-P1456274-4	-	97	92	92	97	97	101	102	97	95	74
	P495B	10%	QA1975	94	-	93	-	92	-	80	79	79	84	57
	P495C	1%	RA0215-P1536458-5	-	-	-	-	-	-	98	99	96	92	37
MEXF UACC-257	P495A	10%	QA1489-P1456245-7	-	103	101	113	101	104	101	109	107	106	68
	-	-	-	-	-	-	-	-	-	-	-	-	-	-
	P495C	1%	RA0216-P1535660-4	-	-	-	-	-	-	101	106	107	77	41
MMX RPMI8226	P495A	10%	QA1491-P1456274-5	-	86	80	76	83	84	89	87	98	99	10
	P495B	10%	QA1974	100	-	100	-	100	-	88	92	94	95	16
	P495C	1%	RA0218-P1537021-5	-	-	-	-	-	-	102	101	95	77	5
OVXF OVCAR-8	P495A	10%	QA1490-P1456251-6	-	84	90	86	85	86	84	92	90	96	67
	P495B	10%	QA1972	92	-	90	-	86	-	89	84	82	74	32
	P495C	1%	RA0216-P1535660-5	-	-	-	-	-	-	102	104	100	61	22
PRXF PC-3	P495A	10%	QA1490-P1456251-7	-	91	83	87	87	91	86	89	86	82	52
	P495B	10%	QA1973	81	-	88	-	84	-	89	92	77	74	49
	P495C	1%	RA0217-P1537015-3	-	-	-	-	-	-	102	105	100	58	22

### Effects of PBZ on histone deacetylase activity

**Fig 4:**
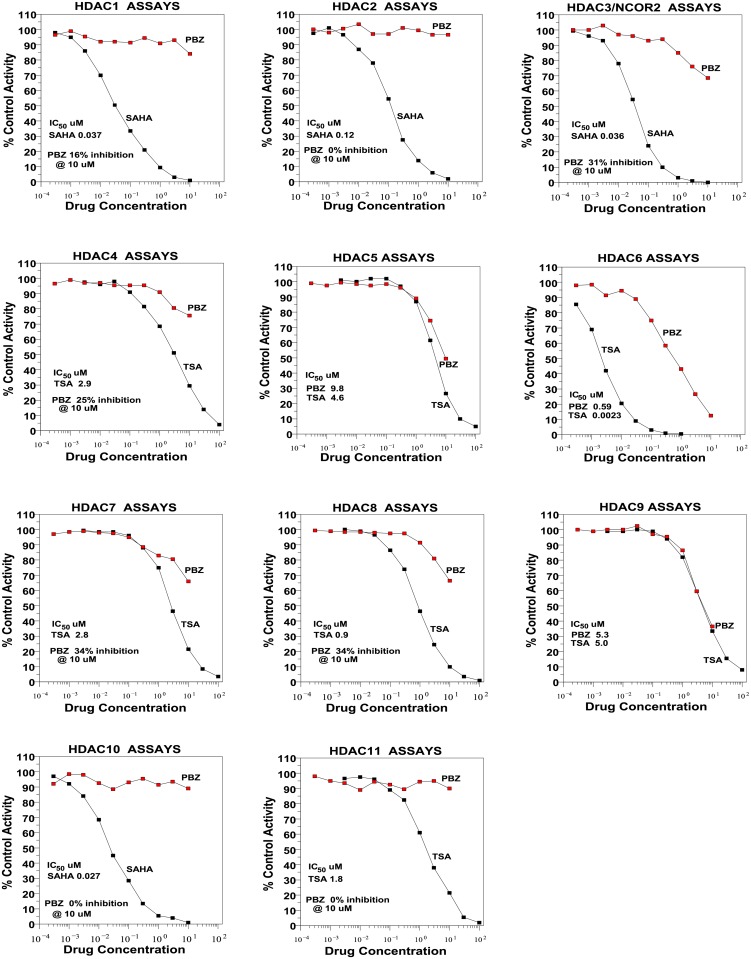
Effects of PBZ on the activity of HDACs 1 thru 11.

The inhibitory activity of PBZ on HDACs 1 thru 11 are presented in Fig 4.

**Fig 5:**
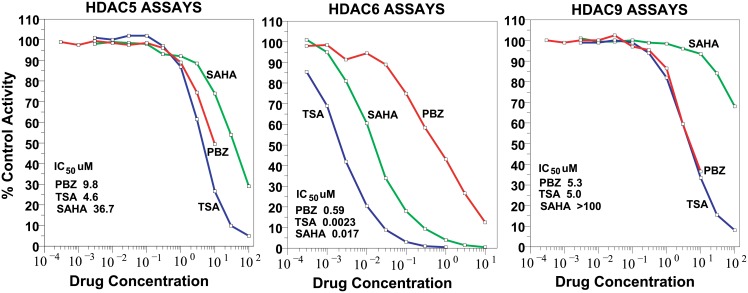
Comparison of the inhibitory activities of PBZ on HDACs 5, 6. and 9 with those for TSA and SAHA.

HDAC6 was the most sensitive to PBZ, with an IC_50_ value of 0.59 μM. Next in sensitivity were HDAC9 (IC_50_ 5.3 μM) and HDAC5 (IC_50_ 9.8 μM). These activities are also presented in Fig 5 together with the inhibitory activity of TSA and SAHA for the same enzymes. HDACs 3, 7 and 8 showed inhibitions in the 30% range at 10 μM concentrations of PBZ.

## Discussion

CMap gene expression enrichment values for PBZ relative to HDAC inhibitors for a varied group of gene signatures (Table 2) greatly expanded the initial observation that co-sorted PBZ with HDAC inhibitors for a common gene expression signature resulting from the effects of SAHA, TSA and MS-275 in human breast cancer cells and human bladder cancer cells (Table 1) [19]. Some of the highest PBZ enrichment scores that allowed comparisons with SAHA and TSA enrichments (PBZ scores **0.998, 0.995, 0.994, 0.993, 0.991, 0.986, 0.972, 0.962**) were found in MSigDB for ultraviolet exposure signatures in the low wavelength range, 100 to 280 nm, UVC class radiation. These signatures relate to both trichothiodystrophy (TTD) syndrome and xeroderma pigmentosum (XP) in combination with features of Cockayne syndrome (XP/CS) [21,22]. Despite the fact that these syndromes appear to share certain common defects in nucleotide excision repair competence (NER) there are distinct differences in the clinical manifestations of the syndromes, limiting to some extent the absolute importance of NER in some features. A particular difference is that XP patients show a high frequency of skin cancer, while TTD and CS patients have a low incidence of tumors; also, the CS syndrome includes developmental and neurological abnormalities [21]. In view of the complex genomics of these several syndromes, it is not surprising that there are conflicting reports on the effects of HDAC inhibition on the efficiency of NER among these different but related pathologies [23–27]. In the context of this present study, it is primarily of interest that PBZ shows considerable concurrence with classical HDAC inhibitors in regard to gene enrichment scores for UVC-related gene signatures.

PBZ scores **0.990, 0.982, 0.977, 0.974, and 0.970** in Table 2 provide data on human cytomegalovirus (HCMV) gene signatures that include comparative gene enrichment scores for PBZ, TSA and SAHA. It is quite consistently seen that HDAC inhibition results in an increased permissiveness for infection by HCMV and an increase in viral replication [28,29]. This aspect of histone modification with the use of TSA, SAHA, valproic acid and other inhibitors has been applied *in vitro* and *in vivo* to increase the effectiveness of herpes-based oncolytic cancer therapy [30–32]. It is interesting to speculate that PBZ may share some of this potential therapeutic efficacy.

PBZ scores **0.942 and 0.912** can be compared with the enrichment scores for TSA and SAHA for gene signatures resulting from ultraviolet exposure in the range of 280 to 320 nm, UVB radiation. UVB radiation is considered to have the most energy and DNA damaging potential of solar radiation. Exposure of normal epidermal keratinocytes (NHEK) to UVB radiation produced the two signatures included in these comparisons, and has the potential to proceed to basal cell or squamous cell carcinoma [33]. Of interest in relation to the enrichment scores in Table 2, Simpson et al. [34] demonstrated that TSA, through an increased elaboration of the cell adhesion protein, desmoglein 1,was able to prevent fragmentation of monolayers of keratinocytes induced by an exfoliative bacterial toxin, Also, in a follow up on this work, Johnson et al. [35] demonstrated that TSA was able to increase epidermal desmoglein 1 and help recover markers of keratinocyte differentiation following UVB exposure.

Finally, PBZ scores **0.909 and 0.850** relate to gene signatures from colon cancer cells treated with butyrate, and PBZ **0.823** score for cells treated with TSA. There is an extensive literature on the effects of TSA, SAHA, valproic acid and sodium butyrate on colon cancer cell lines with growth arrest, stimulation of maturation, and induction of apoptosis [36–39]. PBZ enrichment scores for PBZ compared favorably with those of TSA and SAHA for these gene signatures.

The co-sorting of PBZ with other HDAC inhibitors for a number of gene signatures (Tables 1 and 2), and evidence of anti-proliferative effects on human tumor cell cultures (Figs 2 and 3, and Tables 3 and 4) prompted studies that demonstrated HDAC inhibitory activity with PBZ (Figs 4 and 5). A review of the literature through PubMed and other search resources did not reveal any prior report or contention of PBZ as an HDAC inhibitor. However, there is prior evidence of its potential anti-proliferative effects. Gheeya et al. demonstrated that epoxy anthraquinone derivative (EAD) inhibited multiple cell lines of neuroblastoma cells by inhibition of DNA replication through several mechanisms. Using CMap analysis to identify potential agents with similar activity to EAD, PBZ showed a high gene enrichment score (connectivity) relative to EAD [40]. The authors proposed that this might be related to a potential DNA alkylating activity and/or alpha-adrenergic blockade. Wang et al. applied high-throughput screening of the effects of 1280 small molecules on growth in two human neuroblastoma cell lines [41]. PBZ was one of four compounds that showed dose-dependent inhibition of cell growth, invasion and migration. It was suggested that these effects might be related to the calmodulin inhibitory activity of PBZ or its effects as an alpha- adrenergic antagonist.

Karube et al. [42] used CMap analyses in search of candidate drugs with potential efficacy against NK cell neoplasms (lymphoid cancers). PBZ was rated with a high negative gene enrichment score in their map, suggesting possible therapeutic value in these malignancies. The inhibitory potency of PBZ was considerably lower than that for puromycin, SAHA and TSA in cellular assays; most of the work was therefore centered on SAHA. It is of interest that the drugs, including PBZ, were dissolved and diluted in DMSO; as noted above, DMSO appeared to interfere with PBZ potency in the current work.

Two papers focused on the involvement of PBZ in proliferative processes in the central nervous system. Cobret et al. [43] identified PBZ in a screen of 1263 small molecular weight molecules as having the highest complex formation with LINGO-1, which negatively regulates neuritic growth and survival of neurons. LINGO-1 is found only in neuronal tissue as part of a plasma membrane and endoplasmic reticulum complex with apparent receptor function and molecular signaling properties. Its ligand complex with PBZ enhances its negative regulation of growth and survival [43]. These observations were extended by the recent report of Lin et al. [44] demonstrating anti-proliferative effects of PBZ in U251 and U87MG malignant glioma cell lines. They demonstrated inhibitory effects on proliferation, invasion and migration in *in-vitro* studies, and a marked reduction in expansion of tumor volume in vivo with the direct injection of PBZ into implanted tumors. Western blot analysis showed a marked increase in LINGO-1 expression in U251 cells following PBZ treatment, which may explain its negative proliferative effects.

Lee [45] observed a marked inhibitory effect of PBZ on the cellular proliferative activity of vascular smooth muscle that leads to the development of pulmonary arterial hypertension (PAH) in a rat model. The vascular remodeling primarily involved medial thickening of pulmonary arterioles, with severe narrowing of the vascular channel; the obstructive nature of this remodeling leads to a greatly heightened afterload against right- ventricular outflow, with a resulting right-ventricular hypertrophy and eventual right-heart cardiac failure. The two most common animal models of PAH involve chronic hypoxic ventilation or administration of the pulmonary toxin, monocrotaline. Lee found that daily injections of PBZ following treatment with monocrotaline resulted in a 69% decrease in right-ventricular hypertrophy and an 80% inhibition in the increase in medial thickening of pulmonary arteries after 4 weeks of PBZ treatment. Relevance to the present study is found in that HDAC inhibition has drawn considerable recent consideration in experimental models of PAH, especially in regard to the importance of utilization of specific classes of inhibitors to obtain a net beneficial clinical result [46]. Kim et al. [47] demonstrated that specific HDAC IIa inhibition was able to substantially reverse established PAH in both monocrotoline and hypoxic animal models.

It is not possible to propose clinical translation of the observations in these present studies of an HDAC inhibitory activity of PBZ. However, the examples of anti-proliferative actions of PBZ noted above may warrant interest in this connection. PBZ showed fairly significant inhibitory potential among both Class IIa (HDAC5, HDAC9) and Class IIb (HDAC6) isoforms (Figs 4 and 5). In addition, the inhibition of HDACs 3, 7 and 8 in the 30% range at 10 μM concentration may contribute, in part, to the range of inhibitory activity that was seen in the NCI60 tumor cell culture screen (Fig 2).

The inhibitory potencies of PBZ on HDAC5 (IC_50_ 9.8 μM) and on HDAC9 (IC_50_ 5.3 μM) (Fig 5) were fairly comparable to the potencies of TSA in these assays, i.e., IC_50_ 4.6 μM and IC_50_ 5.0 μM respectively, for TSA. And, PBZ was more potent than SAHA in inhibition of both HDAC 5 and 9 (Fig 5). These results may be of interest in relation to the associations that were identified for PBZ with known HDAC inhibitors in the CMap findings (Tables 1 and 2). High levels of expression of HDAC5 and HDAC9 mRNA and protein in primary pediatric medulloblastoma tumors are associated with poor survival; also, transfection with multiple siRNAs for HDAC5 and 9 in established tumor cell lines caused a reduction in cell proliferation in all of the transfection vectors that were studied [48]. HDAC5 mRNA and protein expression in breast cancer are known to promote metastasis and to be important negative prognosticators of survival [49]. The potency of PBZ on HDAC 5 and 9 (Fig 5) relative to that of other established HDAC inhibitors suggests potential areas for further studies.

PBZ showed the highest potency against HDAC6 under the conditions of these present assays (Figs 4 and 5). This is of interest in relation to the possible association that we and others have found for the use of the drug in neuropathic pain syndromes [2,4,5]. Studies by Krukowski et al. [50] demonstrated that a highly selective HDAC6 inhibitor, ACY-1083, showed a remarkable capacity to both prevent and reverse the peripheral neuropathy induced by treatment with cisplatin in mice. The HDAC6 inhibitor normalized the sensitivity threshold for pain (paw withdrawal), an established measure of mechanical allodynia. Increased acetylation of α- tubulin in peripheral nerve, restoration of mitochondrial energetics in peripheral nerves and dorsal root ganglia, and, with prolonged treatment, restoration of intra-epidermal innervation of the paw were observed. Certain aspects of these results were confirmed in mice with administration of larger doses of a less potent HDAC6 inhibitor, ACY-1215, following a course of treatment with cisplatin.

PBZ may have an advantage in relation to possible new applications in that it has been in clinical use since approval by the FDA in 1953. The drug is only labeled for one use, treatment of paroxysmal hypertension and sweating in patients with a pheochromocytoma. The drug is clinically recognized as appropriate for off-label applications in the treatment of several urinary tract conditions, including benign prostatic hypertrophy, neurogenic bladder, micturation disorders, prostatitis and post-operative urinary retention following epidural administration of morphine [1,51]. In an extensive review of the clinical use of PBZ, Te [51] estimated that over 20,000 patients in the United States alone received PBZ during the period from 1953 to 2002. The drug has relatively mild adverse effects that are expected in relation to its α-adrenergic blocking activity. The more common include nasal congestion, dizziness from postural hypotension accompanied by tachycardia, and miosis. These effects usually decrease with continued therapy. Fatigue, confusion, headache, inhibition of ejaculation, and gastrointestinal symptoms occur with less frequency or rarely [1,51]. PBZ carries a warning of mutagenic and carcinogenic activity, but in its long clinical use there appears to be no established evidence of a cancer risk with this drug at this time [1,5,51]. The minor adverse effect profile of PBZ may have an advantage over some HDAC inhibitors.

In view of the FDA-approved status of the drug, it may be possible to test the drug in an appropriate case where there is a poor clinical prognosis and first-line therapies have been exhausted, or through an FDA Investigational Drug Application for an off-label treatment protocol.

## Conclusions

The patterns of gene expression that were induced by PBZ in several tumor cell lines (BroadBuild02 Molecular Signature Database) suggested that this drug may have HDAC inhibitory activity. Inhibition of HDACs 5, 6, and 9 reached potencies that may suggest a value for further investigations, especially since the drug is already FDA approved and has a long clinical history. The limitations of the present studies are that they did not explore whether the inhibition of certain HDACs by PBZ had a direct relationship to the suppression of tumor growth rates by the drug, nor did they establish a clinical translation. These questions require further investigation.

## Supporting information

S1 Appendix Cell culture details. (DOCX)

S2 Appendix Histone deacetylase assay details. (DOCX)
