# Supplementary material for: Anti-tumor activity of phenoxybenzamine and its inhibition of histone deacetylases
Source: PLoS One. 2018 Jun 13;13(6):e0198514. doi: 10.1371/journal.pone.0198514 (PMC5999115; doi:10.1371/journal.pone.0198514)
Supplement: S1 Appendix — (DOCX) [file pone.0198514.s001.docx]

**S1 Appendix: Cell Culture Details**

**National Cancer Institute (NCI60) screen cultures:**

All of the cultures in the NCI60 screen are available from a repository maintained by Charles River Laboratories under a contract with the National Cancer Institute; the repository catalog can be accessed through the following link: <https://dtp.cancer.gov/organization/btb/docs/DCTDTumorRepositoryCatalog.pdf>.

**Oncotest Laboratories Cell culture Assay Condition Details:**

“Cell lines were routinely passaged once or twice weekly and maintained in culture for up to 20 passages. Cells were grown at 37°C in a humidified atmosphere with 5% CO_2_ in RPMI 1640 medium (25 mM HEPES, with L-glutamine, #FG1385, Biochrom, Berlin, Germany) supplemented with 10% (v/v) fetal calf serum (Sigma, Taufkirchen, Germany) and 0.1 mg/mL gentamicin (Life Technologies, Karlsruhe, Germany).”

**Oncotest tumor cell line panel:**

NCI: National Cancer Institute, Bethesda, MD, USA.

ECACC: European Collection of Authenticated Cell Cultures, Salisbury, UK.

DSMZ: Deutsche Sammlung von Mikroorganismen und Zellculturen, Braunschweig, Germany.
