# Supplementary material for: Anti-tumor activity of phenoxybenzamine and its inhibition of histone deacetylases
Source: PLoS One. 2018 Jun 13;13(6):e0198514. doi: 10.1371/journal.pone.0198514 (PMC5999115; doi:10.1371/journal.pone.0198514)
Supplement: S2 Appendix — (DOCX) [file pone.0198514.s002.docx]

**S2 Appendix: Histone deacetylase assay details:**

**BPS Bioscience Laboratories:**

Assay Conditions:

“The serial dilution of the compound (PBZ) was first performed in PBS buffer with the highest concentration at 100μM. The serial dilution of SAHA and TSA was first performed in 100% DMSO with the highest concentration at 1mM. Each intermediate compound dilution (in 100% DMSO) will then get directly diluted 10x fold into assay buffer for an intermediate dilution of 10% DMSO in HDAC assay buffer and 5µl of the dilution was added to a 50µl reaction so that the final concentration of DMSO is 1% in all of reactions for the control compounds. The compounds were pre-incubated in duplicate 37 ºC for 1 hour in a mixture containing HDAC assay buffer, HDAC enzyme and a test compound . After 1 hour, the enzymatic reactions were initiated by the addition of HDAC substrate to a final concentration of 10μM. The enzymatic reaction proceeded for 30 minutes at 37ºC.

After enzymatic reactions, 50μl of 2 x HDAC Developer was added to each well for the HDAC enzymes and the plate was incubated at room temperature for an additional 15 minutes.

Fluorescence intensity was measured at an excitation of 360 nm and an emission of 460 nm using a Tecan Infinite M1000 microplate reader.

Data Analysis:

HDAC activity assays were performed in duplicates at each concentration. The fluorescent intensity data were analyzed using the computer software, Graphpad Prism. In the absence of the compound, the fluorescent intensity (F_t_) in each data set was defined as 100% activity. In the absence of HDAC, the fluorescent intensity (F_b_) in each data set was defined as 0% activity. The percent activity in the presence of each compound was calculated according to the following equation: %activity = (F-F_b_)/(F_t_-F_b_), where F= the fluorescent intensity in the presence of the compound.

The values of % activity versus a series of compound concentrations were then plotted using non-linear regression analysis of Sigmoidal dose-response curve generated with the equation Y=B+(T-B)/1+10^((LogEC50-X)×Hill Slope)^, where Y=percent activity, B=minimum percent activity, T=maximum percent activity, X= logarithm of compound and Hill Slope=slope factor or Hill coefficient. The IC_50_ value was determined by the concentration causing a half-maximal percent activity.”

**Reagents:**

SAHA purchased from Cayman Chemicals (Ann Arbor, MI, Catalog Number 10009929).

TSA purchased from Selleck (Houston, TX, Catalog number S1045).

HDAC Assay Buffer (BPS catalog number 50031)

HDAC Assay Developer (BPS catalog number 50030)

HDAC Class 2a Substrate 1 (BPS catalog number 50040)

HDAC Substrate 3 (BPS catalog number 50037)

|  | Catalog # | Enzyme Lot # | Enzyme Used (ng) / Reaction | Substrate |
| --- | --- | --- | --- | --- |
| HDAC1 | 50051 | 170105-1 | 7.2 | 10μM HDAC Substrate 3 |
| HDAC2 | 50002 | 130823-G | 7.5 | 10μM HDAC Substrate 3 |
| HDAC3/NCOR2 | 50003 | 130819 | 3.4 | 10μM HDAC Substrate 3 |
| HDAC4 | 50004 | 130828-G | 0.3 | 2μM HDAC Substrate Class 2a |
| HDAC5 | 50005 | 140211 | 44 | 2μM HDAC Substrate Class 2a |
| HDAC6 | 50006 | 170518-1 | 10 | 10μM HDAC Substrate 3 |
| HDAC7 | 50007 | 150629-5 | 1.6 | 2μM HDAC Substrate Class 2a |
| HDAC8 | 50008 | 161216 | 27 | 2μM HDAC Substrate Class 2a |
| HDAC9 | 50009 | 130502-66 | 4.3 | 2μM HDAC Substrate Class 2a |
| HDAC10 | 50060 | 150709-1A | 600 | 10μM HDAC Substrate 3 |
| HDAC11 | 50011 | 160223 | 60 | 2μM HDAC Substrate Class 2a |
